# Auf Solidarität muss Solidität folgen

**DOI:** 10.1007/s41358-022-00317-3

**Published:** 2022-03-15

**Authors:** Berthold Busch, Jürgen Matthes

**Affiliations:** Kompetenzfeld Internationale Wirtschaftsordnung und Konjunktur, Institut der deutschen Wirtschaft Köln e. V., Konrad-Adenauer-Ufer 21, 50668 Köln, Deutschland

**Keywords:** Europäische Währungsunion, Euro-Schuldenkrise, Next Generation EU, Aufbau- und Resilienzpläne, Staatsverschuldung, European monetary union, Euro debt crisis, Next generation EU, Recovery and resilience plans, Government debt

## Abstract

Die Mitgliedstaaten der Europäischen Union (EU) haben sich in den letzten beiden Jahrzehnten in erheblichem Ausmaß solidarisch mit einzelnen Ländern verhalten, die in finanzielle Schwierigkeiten gekommen sind. Solidarität ist keine Einbahnstraße, sie erfordert Gegenseitigkeit. Diese können die Mitgliedstaaten leisten, indem sie auf mehr Solidität in der nationalen Finanz- und Wirtschaftspolitik setzen. Die erste große Solidaritätsaktion reagierte auf die Euro-Schuldenkrise. Trotz wirtschaftspolitischer Fehler und der Tatsache, dass die Krise in dieser Tiefe selbst verursacht war, bewiesen die übrigen EU-Staaten Solidarität. Sie gingen dabei erhebliche finanzielle Risiken für ihre eigenen Steuerzahler ein, indem sie umfassende Unterstützungsmaßnahmen ergriffen und einen Euro-Rettungsfonds etablierten. Solidarisch zeigte sich die EU mit umfangreichen Hilfspaketen auch in der Corona-Pandemie. Auf das erste Corona-Krisenpaket der EU vom Mai 2020 folgte ein als Next Generation EU (NGEU) bezeichnetes Aufbauinstrument. Es umfasst ein Volumen von fast 807 Milliarden Euro. Der größte Teil davon fließt in die Aufbau- und Resilienzfazilität und soll für Investitionen und Reformen in den Mitgliedstaaten der EU eingesetzt werden. Die Auszahlung der Mittel ist an Aufbau- und Resilienzpläne gebunden, die von den Mitgliedstaaten vorgelegt werden müssen und von der Europäischen Kommission bewertet werden. Dass die Länder die Pläne selbst machen und die Europäische Kommission diese nicht vorschreibt, sondern nur prüft, erhöht das sogenannte Ownership und die Umsetzungschancen. Die produktive Nutzung der umfangreichen Hilfsgelder ist als Grundvoraussetzung dafür anzusehen, dass sich die Solidarität auch auf lange Sicht als sinnvoll erweist.

## Abstract (en)

Over the past two decades, the member states of the European Union (EU) have shown to a considerable extent solidarity with individual countries that have experienced financial difficulties. Solidarity is not a one-way street; it requires reciprocity. Member states can do this by focusing on more solidity in national fiscal and economic policies. The first major solidarity action responded to the euro debt crisis. Despite economic policy mistakes and the fact that the crisis was self-inflicted to this depth, the other EU states demonstrated solidarity. In doing so, they took significant financial risks for their own taxpayers by taking comprehensive support measures and establishing a euro rescue fund. The EU also showed solidarity with extensive aid packages during the Corona pandemic. The EU’s first Corona crisis package of May 2020 was followed by a reconstruction instrument known as the Next Generation EU (NGEU). It is worth almost 807 billion euros. Most of it goes to the Reconstruction and Resilience Facility and is to be used for investments and reforms in EU member states. Disbursement of the funds is tied to reconstruction and resilience plans, which must be submitted by the member states and are assessed by the European Commission. The fact that the countries make the plans themselves and the European Commission does not prescribe them, but only checks them, increases the so-called ownership and the chances of implementation. The productive use of the extensive aid funds must be seen as a basic prerequisite for solidarity to prove its worth in the long term.

Die Mitgliedstaaten der Europäischen Union (EU) haben sich in den letzten beiden Jahrzehnten in erheblichem Ausmaß solidarisch mit einzelnen Ländern verhalten, die in finanzielle Schwierigkeiten gekommen sind. Solidarität ist keine Einbahnstraße, sie erfordert Gegenseitigkeit. Dies können die Mitgliedstaaten leisten, indem sie auf mehr Solidität in der nationalen Finanz- und Wirtschaftspolitik setzen. Es geht darum, mit Blick auf eine stabilitätsorientierte Politik die „eigenen Hausaufgaben“ zu machen und so den unverzichtbaren eigenen Beitrag zur Stabilität und wirtschaftlichen Stärke der EU zu leisten. Solidarität und Solidität können dann, aber auch nur dann Hand in Hand gehen. Dazu sollten sich die Mitgliedstaaten stärker an der Maßgabe von Artikel 121 des Vertrags über die Arbeitsweise der EU (AEUV) orientieren, ihre Wirtschaftspolitik als eine Angelegenheit von gemeinsamem Interesse zu betrachten. Diese These wird im Folgenden näher erörtert, vor allem mit einem Fokus auf die Europäische Wirtschafts- und Währungsunion (EWU). Denn besonders hier findet sich ein Kristallisationspunkt der Debatte um Solidarität versus Solidität.

Die Einführung einer gemeinsamen Währung im Jahr 1999 – 2002 folgte die Einführung des Euro als Bargeld – war eine umstrittene ökonomische Entscheidung, weil die Geldpolitik vereinheitlicht wurde, die Finanzpolitik aber im Wesentlichen in nationaler Verantwortung blieb. Befürworter sahen im Euro eine Weiterentwicklung des gemeinsamen Binnenmarktes, weil damit Wechselkursänderungen zwischen den Euroländern ausgeschlossen wurden, die den innergemeinschaftlichen Handel verzerren konnten. Gegner der Währungsunion verwiesen darauf, dass den teilnehmenden Staaten die außenwirtschaftliche Anpassung verwehrt würde, die im Fall von Leistungsbilanzungleichgewichten notwendig sei. Die Befürworter der Währungsunion sahen in ihr den Grundstein für das wirtschaftliche Zusammenwachsen der Mitgliedstaaten, die Gegner vertraten die Krönungstheorie (Belke 2020, S. 754), nach der zuerst die Konvergenz der Volkswirtschaften herzustellen sei, auf die dann sozusagen als Krone die gemeinsame Währung folgen sollte. In verschiedenen Manifesten wurden die unterschiedlichen Positionen zum Ausdruck gebracht (Hrbek 1992).

Hinter dieser Debatte stand die Wahrnehmung, dass es unter den Ländern der EWU unterschiedliche Präferenzen für Solidität und Stabilität gab. Erfahrungsgemäß hatte sich dabei in der Vergangenheit eine gewisse Unterscheidung zwischen Ländern im Norden und im Süden Europas gezeigt. Dies war nicht zuletzt darin zum Ausdruck gekommen, dass die Währungen der meisten südeuropäischen Staaten vor der Konvergenzphase auf die EWU hin immer wieder gegenüber der D‑Mark abwerten mussten, weil Löhne und Preise dort stärker gestiegen waren als in Deutschland. Dazu hatte auch eine expansivere Finanzpolitik mit tendenziell höheren öffentlichen Defiziten beigetragen, die in Italien und Griechenland auch zu hohen Staatsschuldenquoten von über 100 % zur Mitte der 1990er-Jahre geführt hatte. Vor diesem Hintergrund forderten viele Länder im Norden, dass bei weiterhin nationaler Verantwortung für die Finanzpolitik verbindliche gemeinsame Regeln für eine hinreichende Stabilitätsorientierung eingeführt werden. So hatte beispielsweise der Deutsche Bundestag im Zusammenhang mit dem Zustimmungsgesetz zum Vertrag von Maastricht darauf verwiesen, dass die Währungsunion auf die haushalts- und finanzpolitische Solidität der teilnehmenden Mitgliedstaaten gründet (Deutscher Bundestag 1992). Und besonders auf deutsche Initiative hin wurde 1997 der Stabilitäts- und Wachstumspakt (SWP) vereinbart, mit dem die fiskalische Solidität abgesichert werden sollte.

Schon in der ersten Dekade der EWU zeigten sich die befürchteten Probleme in Südeuropa, auch wenn sie zunächst von einer vermeintlich erfreulichen wirtschaftlichen Entwicklung dort überdeckt blieben. Die Mitgliedschaft im Euroraum und bereits die Aussicht darauf führten zu einem Rückgang der Nominal- und Realzinsen in Italien, Griechenland, Portugal und Spanien, vor allem weil Währungsabwertungsrisiken nicht mehr bestanden und die Europäische Zentralbank (EZB) mit ihrer zentralisierten Geldpolitik den Glaubwürdigkeitsbonus der Deutschen Bundesbank weitgehend übernehmen konnte. Niedrigere Zinsen kurbelten die Wirtschaft in Südeuropa an. Doch diese Entwicklung war zumindest partiell auf Sand gebaut. Vor allem in Griechenland, Spanien und Portugal kam es zu makroökonomischen Ungleichgewichten und zunehmend auch zu Überhitzungserscheinungen, weil die niedrigeren Zinsen einen Kreditboom in der Privatwirtschaft (vor allem in Spanien und Irland auch im Immobiliensektor) entfachten und auch in den öffentlichen Haushalten Luft für höhere Staats- und vor allem Sozialausgaben verschafften. Besonders in Griechenland, teils auch in Portugal und etwas weniger in Italien blieben trotz geringerer Zinsausgaben die Fiskaldefizite deutlich zu hoch. Diese Entwicklung schlug sich (außer in Italien, dafür aber auch in Irland) in hohen Leistungsbilanzdefiziten nieder, ein Zeichen dafür, dass Südeuropa in weiten Teilen über seine Verhältnisse und auf Pump lebte (SVR 2010/2011, Ziffer 122, 125; Busch et al. 2011a, b).

## Euro-Schuldenkrise

Diese Ungleichgewichte traten nach der globalen Wirtschafts- und Finanzkrise 2008/2009 mit Macht zutage (ERH 2020) und waren eine wesentliche Mitursache für die Schwere der Euroschuldenkrise zwischen den Jahren 2010 und 2013. Sie waren ein Zeichen dafür, dass es fundamental an Solidität der Wirtschaftspolitik gefehlt hatte. Trotzdem zeigte sich Nordeuropa in der Euro-Schuldenkrise solidarisch. Die ersten zehn Jahre waren für die Währungsunion also nur oberflächlich betrachtet eine vergleichsweise ruhige Periode.

Besonders eklatant war die Entwicklung der Finanzpolitik in Griechenland. Nach dem Regierungswechsel im Jahr 2009 wurde offenbar, dass das griechische Haushaltsdefizit viel zu niedrig angegeben worden war. Die neue Regierung korrigierte das Staatsdefizit für das Jahr 2009 zunächst auf 12,7 % des BIP nach oben (Zervakis 2011, S. 373). Eurostat hat später den Wert sogar mit über 15 % angegeben. Die Finanzmärkte reagierten mit einem starken Anstieg der Zinsen für griechische Staatsanleihen; dem Land drohte die Zahlungsunfähigkeit, weil die Schuldentragfähigkeit infrage gestellt war. Griechische Anleihen wurden auf das Niveau von Junk Bonds herabgestuft (Zervakis 2011, S. 373).

Anfang Mai 2010 spitzte sich die Lage weiter zu, denn es kam zu Ansteckungseffekten vor allem auf andere Länder in der sogenannten Peripherie der Währungsunion (Weber 2010, S. 10). Betroffen waren zunächst besonders die Länder mit den hohen makroökonomischen Ungleichgewichten und Leistungsbilanzdefiziten. Auf Italien sprang die Krise erst 2011 über, unter anderem, weil im Zuge einer drohenden Umschuldung und Privatgläubigerbeteiligung in Griechenland auch die hohen italienischen Staatsschulden Sorge bereiteten. Zudem hatte der damalige Ministerpräsident Berlusconi den italienischen Finanzminister Tremonti für seine vermeintlich zu zurückhaltende Fiskalpolitik kritisiert, was das Vertrauen an den Finanzmärkten weiter erschütterte.

Trotz der aufgezeigten wirtschaftspolitischen Fehler und der Tatsache, dass die Krise in dieser Tiefe selbst verursacht war, bewiesen die übrigen EU-Staaten Solidarität. Sie gingen dabei erhebliche finanzielle Risiken für ihre eigenen Steuerzahler ein, indem sie umfassende Unterstützungsmaßnahmen ergriffen und einen Euro-Rettungsfonds etablierten. Darüber hinaus nahm die EZB für das Finanzsystem die Rolle des Lender of Last Resort ein (siehe Abschnitt *Hilfspakete in der Euro-Schuldenkrise*). Diese Hilfsbereitschaft war aber auch dem Ausmaß der Krise geschuldet, in der sogar Zweifel am Bestand der Währungsunion insgesamt aufgekommen waren. Zudem erschien es anders als zuvor erwartet plötzlich möglich, dass europäischen Industrieländern ein Staatsbankrott drohen und durch die bedrohliche Lage der Staatsschulden in Griechenland die EWU insgesamt bedroht sein könnte. Daher waren umfangreiche und entschiedene Hilfsmaßnahmen nötig, um den Finanzmärkten wieder Vertrauen zu geben.

Eine Bewertung dieser Hilfsmaßnahmen muss differenziert erfolgen. Aufgrund der hohen Risiken für die europäischen Steuerzahler können die Rettungsschirme durchaus kritisch gesehen werden. Wäre vorher eine solide Wirtschafts- und Finanzpolitik im Sinne des Maastricht-Vertrags und des SWP betrieben worden, hätte eine so tiefe Krise zweifellos vermieden werden können. Denn anders als vor allem Deutschland, Frankreich und einige andere EU-Länder waren die südeuropäischen Staaten weniger von der globalen Finanzmarkt- und Wirtschaftskrise betroffen. Zudem wurde mit den Hilfsmaßnahmen auf den ersten Blick die No-Bailout-Regel ausgehebelt und die Finanzpolitik der Mitgliedstaaten zumindest teilweise der Disziplin der Finanzmärkte entzogen (Hattenberger 2019, S. 1997). Die Schwere und Tragweite der Krise sowie die plötzliche massive Reaktion und das Herdenverhalten der Finanzmärkte in den Jahren nach 2010 ließen diese Maßnahmen nötig erscheinen. Einen Staatsbankrott Griechenlands zu riskieren, wie zuweilen gefordert wurde, hätte möglicherweise nur schwer kalkulierbare gravierende Folgen gehabt. Auch wenn dies ordnungspolitisch die richtige Maßnahme gewesen wäre, muss eine verantwortungsvolle Politik derartig große und schwer kalkulierbare Risiken vermeiden.

Zudem wurde bei den Rettungsmaßnahmen das Prinzip „Hilfe gegen Reformen“ etabliert. Die finanziellen Unterstützungsmaßnahmen wurden nur unter der Bedingung vergeben, dass die Empfängerstaaten umfangreiche Reformen in der Wirtschafts- und Finanzpolitik ergriffen, um die zuvor erfolgten Fehler in diesen Bereichen zu adressieren. Mit diesem Ansatz ist ein partieller Verlust der nationalen Souveränität verbunden. Mit dieser Androhung sollen – ganz im Sinne der No-Bailout-Regel – Fehlanreize für eine unsolide Wirtschaftspolitik vermieden werden. Insgesamt war dieser Ansatz erfolgreich (Matthes 2015), nicht zuletzt da bis auf Griechenland die Empfängerstaaten den Rettungsschirm nach drei Jahren wie geplant verlassen konnten. Die Reformprogramme wurden zwar teilweise als harsch und oktroyierend kritisiert, doch waren sie letztlich der Reflex auf die zuvor gemachten wirtschaftspolitischen Fehler und gleichzeitig die Kur dafür. Die Kritik an vermeintlich überzogener fiskalpolitischer Austerität erscheint überzogen, da bei massiven öffentlichen Defiziten eine Sparpolitik letztlich unvermeidbar ist. Fehlsteuerungen im ersten Griechenland-Programm wurden im weiteren Verlauf korrigiert. Die Reformvorgaben lassen sich auch so interpretieren, dass der Solidarität der nordeuropäischen Staaten eine höhere Solidität entgegengesetzt wurde.

### Hilfspakete in der Euro-Schuldenkrise:

Die Hilfspakete folgten in enger zeitlicher Reihenfolge ab Frühjahr 2010 und waren unterschiedlich konzipiert, auch weil die Struktur der Rettungsmaßnahmen erst errichtet werden musste:

- Den Anfang machte im April 2010 ein Programm für Griechenland, das neben IWF-Krediten bilaterale Kredite der Eurostaaten in Höhe von insgesamt 80 Mrd. € enthielt.
- Es folgte Anfang Mai 2010 der sogenannte Rettungsschirm für den Euroraum, der aus drei Säulen bestand (Hattenberger 2019, S. 1996 f.)
  - Die erste Säule war der Europäische Finanzstabilisierungsmechanismus (EFSM) mit einem Kreditvolumen von 60 Mrd. €, das über den EU-Haushalt abgesichert wurde.
  - Die zweite Säule war die Gründung einer befristeten Zweckgesellschaft, die Europäische Finanzstabilisierungsfazilität (EFSF), die Kredite in einem Gesamtumfang von 440 Mrd. € vergeben konnte; abgesichert durch bilaterale Haftungszusagen der Eurostaaten (Hattenberger 2019, S. 1997). Die EFSF wurde später durch den Europäischen Stabilitätsmechanismus (ESM) als dauerhafte Einrichtung abgelöst. Der ESM verfügt über ein Stammkapital von 700 Mrd. €; davon sind 80 Mrd. direkte Einlagen und 620 Mrd. in Form von Garantien. Damit soll der ESM Kredite in Höhe von 500 Mrd. € am Kapitalmarkt aufnehmen und an Krisenländer vergeben können.
  - Als dritte Säule kamen wiederum IWF-Kredite hinzu.

**Übersicht über Hilfen nach Ländern**

- Die finanzielle Unterstützung auf der Grundlage der genannten Maßnahmen betrug für Griechenland insgesamt knapp 257 Mrd. €; hinzu kamen noch IWF-Kredite in Höhe von 32,3 Mrd. €.
- Portugal wurde vom EFSM und der EFSF mit 50,3 Mrd. € Krediten unterstützt; zusätzlich kamen vom IWF 25,7 Mrd. €.
- Spanien nahm 41,3 Mrd. € vom ESM in Anspruch, die Zusage lag sogar bei 100 Mrd. €.
- An Irland wurden 40,2 Mrd. € Kredite vergeben, vom IWF kamen zusätzlich 22,6 Mrd. € sowie bilaterale Kredite von Dänemark, Schweden und dem Vereinigten Königreich von insgesamt 4,8 Mrd. €.
- Zypern bekam 6,3 Mrd. € ESM-Kredite (Angerer und Dias 2021, S. 10).
- Rumänien als Nicht-Euro-Staat erhielt von 2009 bis 2011 ebenfalls Kredite von EU, IWF, Weltbank, EIB und EBRD, die hier nicht näher betrachtet werden sollen. Ausgezahlt wurden aus der Zahlungsbilanzhilfe der EU 5 Mrd. € (Angerer und Dias 2021, S. 10).
- Ungarn mit 5,5 Mrd. € und Lettland mit 2,9 Mrd. € wurden ebenfalls aus Mitteln der Zahlungsbilanzfazilität gefördert (ERH 2020, S. 50, 2015, S. 18).

Der Europäische Rechnungshof (ERH) beziffert die finanziellen Hilfen, die insgesamt bereitgestellt wurden, auf fast 415 Mrd. € (ERH 2020, S. 76).

Darüber hinaus profitierten die südeuropäischen Staaten auch und gerade von den Unterstützungsleistungen der EZB in Form verschiedener Ankaufprogramme, umfangreicher Stützungsmaßnahmen für die Banken der Krisenländer und den Kreditmöglichkeiten des europäischen Zahlungsverkehrs (Target2). Auch in diesem Rahmen wurden indirekt erhebliche finanzielle Risiken auf europäische Steuerzahler übertragen.

## Corona-Pandemie

Solidarisch zeigte sich die EU mit umfangreichen Hilfspaketen (vgl. Abschnitt *Das erste Corona-Krisenpaket der EU*) auch in der Corona-Pandemie, die sich Anfang 2020 auch in Europa ausbreitete.

### Das erste Corona-Krisenpaket der EU:

Zunächst beschloss die EU im Mai 2020 kurz nach Beginn der Pandemie ein 540 Mrd. € schweres Krisenpaket, das aus drei Teilen besteht (Lehwald und Berner 2020; Müller 2020, S. 117 f.):

- Erstens eine finanzielle Unterstützung von Kurzarbeitsmaßnahmen in den Mitgliedstaaten (SURE = **S**upport to mitigate **U**nemployment **R**isks in an **E**mergency) in Höhe von 100 Mrd. €. Mit der letzten Auszahlung im Mai 2021 waren fast 90 Mrd. € in Anspruch genommen worden. Auf Italien entfielen 27,4 Mrd. €; auf Spanien: 21,3 Mrd. € und auf Griechenland: 5,3 Mrd. € (European Commission 2021a, b).
- Zweitens wurde der Europäische Stabilitätsmechanismus (ESM) mit einer speziellen Kreditlinie mit erweiterten Bedingungen ausgestattet. Das Pandemic Crisis Support Instrument (PCSI) im Umfang von bis zu 240 Mrd. € kann Hilfskredite von 2 % des jeweiligen BIP der 19 Eurostaaten leisten. Unterstützt werden sollen gesundheitspolitische Maßnahmen. Die Kreditlinie ist bislang noch nicht in Anspruch genommen worden.
- Drittens hat die Europäische Investitionsbank (EIB) einen Garantiefonds in Höhe von bis zu 200 Mrd. € eingerichtet, der Liquiditätskredite besonders an kleine und mittlere Unternehmen vergeben kann.

Darüber hinaus einigten sich die Mitgliedstaaten im Juli 2020 auf ein als Next Generation EU (NGEU) bezeichnetes Aufbauinstrument. Dies geschah im Zusammenhang mit den Verhandlungen über den Mehrjährigen Finanzrahmen (MFR) für die Jahre 2021 bis 2027. NGEU kann als ein schuldenfinanzierter Sonderhaushalt gesehen werden, mit dem die EU auf solidarische Finanzhilfe setzt (Schorkopf 2020, S. 22 f.) und umfasst insgesamt ein Volumen von 806,9 Mrd. € in jeweiligen Preisen (European Commission 2021c, S. 8) – die häufig genannte Zahl von 750 Mrd. € ist die Angabe in Preisen von 2018. Der größte Teil davon fließt in die Aufbau- und Resilienzfazilität (723,8 Mrd. €) und soll für Investitionen und Reformen in den Mitgliedstaaten der EU eingesetzt werden. Dazu können die Mitgliedstaaten Kredite, die von der Kommission am Finanzmarkt aufgenommen werden, in Höhe von 385,8 Mrd. € in Anspruch nehmen und sie erhalten ferner Zuschüsse in Höhe von 337,9 Mrd. €. Gut 83 Mrd. € des NGEU-Instruments tragen zu anderen Programmen der EU bei, worunter 50,6 Mrd. € allein auf das React-EU-Programm entfallen, ein kohäsionspolitisches Instrument.

Die Aufteilung der Mittel auf die Mitgliedstaaten erfolgt nach einem festgelegten Schlüssel, mit dem zunächst 70 % der Zuschüsse bis zum 31. Dezember 2022 und die übrigen 30 % bis zum 31. Dezember 2023 gebunden werden können. Die endgültige Aufteilung auf die Länder wird erst im Jahr 2022 feststehen (SVR 2021/2022, Tz. 190). Die Auszahlungen der Zuschüsse und der Darlehen sollen bis Ende 2026 erfolgen. Gut 50 % der Zuschüsse gehen nach Südeuropa: Spanien, Italien, Portugal und Griechenland. Diese vier Länder bekommen auch pro Kopf die höchsten Mittel aus NGEU, wenn man einmal von Kroatien absieht (Sapała und Thomassen 2021, S. 3). Gemessen an den gesamten Staatsausgaben (2019) fließen die meisten Mittel nach Griechenland, Italien und Kroatien (Sapała und Thomassen 2021, S. 10). Die Darlehen sind auf maximal 6,8 % des nationalen Bruttonationaleinkommens begrenzt. Allerdings werden nicht alle EU-Staaten Kredite in Anspruch nehmen. Aktuell haben nur Griechenland, Italien und Rumänien den maximalen Kreditbetrag beantragt, vier weitere Länder wollen den Kreditplafonds nur zum Teil ausschöpfen. Bliebe es dabei, würden nur 166 Mrd. € kreditiert.

NGEU wird – im Übrigen auch im Koalitionsvertrag der neuen Bundesregierung – als einmaliges, temporäres Aufbauinstrument bezeichnet (Stöcklin 2021, S. 31). Diese Ansicht ist allerdings nicht unumstritten. Es wird befürchtet, dass der Wiederaufbaufonds zu einer Dauerlösung werden kann (Bundesrechnungshof 2021, S. 4). Die Tilgung von der EU aufgenommener Kredite ist bis zum Jahr 2058 geplant. Aus welchen Quellen dies erfolgen soll, ist noch nicht genauer festgelegt. Inzwischen hat die Europäische Kommission die Einführung neuer Eigenmittel vorgeschlagen (Europäische Kommission 2021).

Voraussetzung für die Auszahlung der Hilfsgelder sind Aufbau- und Resilienzpläne, die von den Mitgliedstaaten vorgelegt werden müssen und von der Europäischen Kommission bewertet werden. Auf Vorschlag der Kommission erfolgt eine Billigung durch den Rat der EU. Die unterstützten Maßnahmen sollen zu mindestens 37 % zum ökologischen Wandel und zu mindestens 20 % zur Digitalisierung beitragen. Mit der erklärten digitalen und ökologischen Transformation scheint die EU ein neues Narrativ gefunden zu haben, ähnlich dem Narrativ der Vollendung des Binnenmarktes in den 1980er-Jahren und dem der Schaffung einer Währungsunion in den 1990er-Jahren.

Nach Schätzungen der Europäischen Kommission gibt es die größten Wachstumseffekte in Griechenland, wo das BIP im Jahr 2026 von 2,1 bis 3,3 % höher ausfallen könnte. Für Italien und Spanien wird ein Plus von bis zu 2,5 % geschätzt, für Portugal ein Plus von bis zu 2,4 % (Dias et al. 2021, S. 6). Berücksichtigt man zusätzlich länderübergreifende Effekte, ergeben sich noch etwas höhere Werte. Neben der Absorptionsfähigkeit der Mitgliedstaaten hängt der zusätzliche Wachstumsimpuls vor allem davon ab, ob zusätzliche Maßnahmen finanziert werden (SVR 2021/2022, Tz. 193).

Wenn man die Krisenpakete in der Corona-Krise und der Euro-Schuldenkrise vergleicht, lassen sich dabei Parallelen und Unterschiede erkennen. Der wichtigste Unterschied ist, dass die direkte Krisenursache, anders als bei der Griechenlandkrise, nicht selbst verschuldet war. Vielmehr war die Pandemie ein externer Schock, der alle Länder traf. Doch die wirtschaftlichen Effekte waren unterschiedlich gravierend. Das lag nicht nur an den Unterschieden bei der Schwere der Pandemie-Auswirkungen, sondern auch bei der Solidität der Staatsfinanzen vor der Krise. In der Folge kam es in einigen Ländern zu Beeinträchtigungen bei der fiskalischen Reaktionsfähigkeit auf die Corona-Krise und – verschärft noch durch den krisenbedingten Schuldenanstieg – auch bei dem zukünftigen staatlichen Handlungsspielraum, der in den kommenden Jahren entscheidend sein wird für die Bewältigung der großen transformatorischen Herausforderungen in Sachen Klimaschutz und Digitalisierung.

Einige EU-Länder wiesen vor der Pandemie schon hohe Staatsschuldenquoten auf und hatten diese nicht durch eine angemessene Konsolidierung in den guten Jahren vor der Krise zurückgeführt. Nimmt man die Werte von 2019, dem letzten Jahr vor der Covid-19-Pandemie, so lag die Verschuldung des Staates in Griechenland mit 180,7 % des BIP am höchsten, gefolgt von 134,3 % in Italien. Portugal kam auf 116,6 % und Spanien auf 95,5 %. Gegenüber 2008, dem Jahr, in dem die Finanzkrise begann, hatte Griechenland um mehr als 70 Prozentpunkte zugelegt, Spanien um fast 56 und Italien um 28; Portugal um 44 Prozentpunkte. Besonders bedenklich ist, dass Italien und Frankreich nach der Finanzkrise ihre öffentlichen Schuldenstandsquoten nicht wieder zurückgeführt haben.

Zerlegt man die Veränderung der Schuldenstandsquote, also den Anteil der Staatsverschuldung am Bruttoinlandsprodukt, in ihre verschiedenen Komponenten, zeigt der Vergleich zwischen Frankreich, Spanien und Italien für den Zeitraum 2008 bis 2019 einen deutlichen Unterschied (Busch und Kauder 2021, S. 29): Aufgrund des schwachen Wirtschaftswachstums hat sich die italienische Schuldenstandsquote verschlechtert, während in Spanien vor allem die Finanzpolitik nicht solide war. Die Mittel von NGEU sollten daher von Italien für Investitionen genutzt werden, um das Wachstumspotenzial der Volkswirtschaft zu stärken und die notwendigen Reformen, beispielsweise im Justizwesen, zu ergreifen. Spanien muss neben Reformen vor allem auf mehr fiskalische Solidität setzen. Auch in Frankreich ist mehr fiskalische Disziplin gefordert. Es erscheint daher zumindest fragwürdig, wenn der französische Staatspräsident (zusammen mit dem italienischen Ministerpräsidenten) für eine Aufweichung der Schuldenregeln plädiert (Draghi und Macron 2021).

In der Bewertung ist zunächst hervorzuheben, dass sich die EU mit dem ersten Krisenpaket als schnell handlungsfähig erwies und sie mit dem NGEU-Fonds zu Recht die mittelfristige Perspektive in den Blick nahm. Denn ohne europäische Unterstützung würde es den hochverschuldeten EU-Ländern aufgrund der Schwächung durch die Corona-Krise schwerfallen, die nötigen staatlichen Mittel aufzubringen, um die beiden anstehenden Transformationen zu meistern. Daher erscheint die wieder geübte Solidarität grundsätzlich gerechtfertigt, auch wenn es in einigen Ländern zuvor erneut an wirtschaftspolitischer Solidität mangelte. Ähnlich wie in der Euro-Schuldenkrise setzen die Aufbau- und Resilienzpläne an der Wirtschaftspolitik an. Sie sind aber deutlich stärker auf Investitionen als auf Reformen ausgerichtet. Das ist zwar teils gerechtfertigt, weil alle EU-Länder einbezogen sind und der Fokus nicht auf einer Bekämpfung der vorherigen wirtschaftspolitischen Fehler liegt. Gleichwohl wäre beispielsweise in Italien ein stärkerer Fokus auf Schwachstellen am Arbeitsmarkt und im Bildungssystem sinnvoll gewesen, um das chronisch schwache Wachstum anhaltend zu beleben (Matthes 2021). Ein guter Ansatz ist es zudem, dass die Länder die Pläne selbst machen und die Europäische Kommission diese nicht vorschreibt, sondern nur prüft. Das erhöht das sogenannte Ownership und die Umsetzungschancen. Die produktive Nutzung der umfangreichen Hilfsgelder ist als Grundvoraussetzung dafür anzusehen, dass sich die Solidarität auch auf lange Sicht als sinnvoll erweist.

Hier bestehen allerdings einige Risiken. Diese betreffen zum einen vor allem die politische Umsetzung der angekündigten Reformen. Hier ist eine genaue Überwachung durch die Europäische Kommission und auch durch unabhängige Institutionen und Stakeholder nötig. Zudem steht die sogenannte Absorptionsfähigkeit infrage. Bedeutsam für die Wirksamkeit der Mittel ist der zeitnahe Abruf. In der Kohäsionspolitik weisen die Mitgliedstaaten deutliche Unterschiede auf, wenn man die Ausschöpfung der Mittel aus den Strukturfonds für die Periode 2014 bis 2020 als Maßstab heranzieht. Ende des Jahres 2020 hatten die EU-Staaten insgesamt nur 55 % der zugeteilten Beträge ausgeschöpft, die noch nicht ausgeschöpften Mittel summierten sich auf 208,6 Mrd. €. Besonders gering war die Quote in Italien mit 44 %, Spanien kam auf 45 % – also den beiden Ländern, die jeweils fast 70 Mrd. € als Zuschüsse aus der Aufbau- und Resilienzfazilität erhalten dürften. Besonders hoch lagen die Ausschöpfungsquoten Ende 2020 in Finnland mit 79 % und Irland mit 77 % (ERH 2021, S. 78). Italien und Spanien müssen ihre Absorptionsfähigkeiten verbessern, um die Mittel aus der Aufbau- und Resilienzfazilität auszuschöpfen.
